# Snakebite: An Exploratory Cost-Effectiveness Analysis of Adjunct Treatment Strategies

**DOI:** 10.4269/ajtmh.17-0922

**Published:** 2018-06-04

**Authors:** Benjamin J. Herzel, Stephen P. Samuel, Tommaso C. Bulfone, C. Soundara Raj, Matthew Lewin, James G. Kahn

**Affiliations:** 1 Philip R. Lee Institute for Health Policy Studies, University of California, San Francisco, San Francisco, California;; 2 Loma Linda University School of Medicine, Loma Linda, California;; 3 Queen Elizabeth Hospital, King’s Lynn, United Kingdom;; 4 California Academy of Sciences, San Francisco, California;; 5 Ophirex, Inc., Corte Madera, California;; 6 TCR Multispecialty Hospital, Krishnagiri, Tamil Nadu, India

## Abstract

The cost-effectiveness of the standard of care for snakebite treatment, antivenom, and supportive care has been established in various settings. In this study, based on data from South Indian private health-care providers, we address an additional question: “For what cost and effectiveness values would adding adjunct-based treatment strategies to the standard of care for venomous snakebites be cost-effective?” We modeled the cost and performance of potential interventions (e.g., pharmacologic or preventive) used adjunctively with antivenom and supportive care for the treatment of snakebite. Because these potential interventions are theoretical, we used a threshold cost-effectiveness approach to explore this forward-looking concept. We examined economic parameters at which these interventions could be cost-effective or even cost saving. A threshold analysis was used to examine the addition of new interventions to the standard of care. Incremental cost-effectiveness ratios were used to compare treatment strategies. One-way, scenario, and probabilistic sensitivity analyses were conducted to analyze parameter uncertainty and define cost and effectiveness thresholds. Our results suggest that even a 3% reduction in severe cases due to an adjunct strategy is likely to reduce the cost of overall treatment and have the greatest impact on cost-effectiveness. In this model, for example, an investment of $10 of intervention that reduces the incidence of severe cases by 3%, even without changing antivenom usage patterns, creates cost savings of $75 per individual. These findings illustrate the striking degree to which an adjunct intervention could improve patient outcomes and be cost-effective or even cost saving.

## INTRODUCTION

The burden of disease due to snakebite in developing countries is high and poorly recognized.^1–4^ Globally, an estimated 125,000 deaths per year are attributable to snakebite.^5,6^ The Million Deaths Study in India estimated 45,900 deaths attributable to snakebite in India in 2005, which translates to one in 200 deaths (even though governmental reporting is much lower).^7^ There is a strong negative correlation across countries between gross domestic product per capita and incidence of snakebite.^8^ Rural farmers are at the greatest risk because of time spent in the fields, where they often incur snakebites on the feet, legs, or hands.^8–10^ The limb deformities and amputations caused by snakebite lead to life-altering disabilities, which in turn contribute to the cycle of poverty.^11^ Adding to these factors are the prohibitively high costs of the current treatments for snakebite—advanced hospital care and antivenom.^8,10^ To address these issues, the World Health Organization (WHO) included snakebite on its list of neglected tropical diseases in 2017.^12^

Patients who receive antivenom in the presence of supportive care are not likely to die of snakebite, but currently, such treatments can only be given in a clinic or hospital setting and it can be prohibitively expensive. Many victims of snakebite do not survive long enough to reach definitive treatment, either because of geographical or economic constraints that can cause morbidity and mortality through delays in care. In India and Nepal, more than 75% of snakebite deaths occur outside the hospital setting.^3,10^ A household survey in Tamil Nadu, India, reported a 9% case fatality rate due to snakebites—and none of these fatalities occurred after the administration of antivenom.^3^ Although still being the most cost-effective treatment available, antivenom remains expensive because the methods for producing antivenom involve snake handling, animal husbandry, and other high-cost steps.^13^ Antivenom costs vary regionally, with total direct costs for treatment as high as $5,700 U.S. Dollars (USD) in India and costs per antivenom vial ranging from $18 to $200 USD in sub-Saharan Africa.^3,13^ Indeed, according to the WHO definition, snakebite treatment is almost always considered a “catastrophic health expenditure.”^3,4^

Several laboratories are working to reduce the cost of antivenom production and develop adjunct therapeutics.^14–29^ Ideally, adjunct treatments could be used outside of the hospital setting, where most deaths occur. The benefits of adjunct pharmacological and/or preventive interventions such as shoes and pressure immobilizations might also be similarly effective. These types of strategies could result in increased health benefits and ultimately decrease overall costs by decreasing the severity of snakebite.

Only two cost-effectiveness analyses on snakebite treatments were identified at the time of our study.^30,31^ These studies have determined that antivenom (administered alongside supportive care) is cost-effective in a variety of West African settings. Our study seeks to answer the following additional question, “For what cost and efficacy values would a combination antivenom/adjunct-based treatment strategy for venomous snakebites be cost-effective when compared with using antivenom alone?”

## METHODS

### Overview and setting.

A decision tree model was constructed in Microsoft Excel Version 15 (Microsoft Corp., Redmond, WA) to understand the health and cost consequences of different treatment strategies for venomous snakebite in southern India. The model was used to analyze and compare the cost-effectiveness of two intervention arms: 1) antivenom and supportive care and 2) an antivenom/adjunct combination strategy with supportive care. A cohort of 100 individuals was modeled from the time of envenomation at age 30 for a time horizon of 43 years (according to the age-adjusted regional life-expectancy for a 30-year-old in India). Envenomation was modeled at age 30 because research has shown that snakebites are most likely to affect individuals between the ages of 20–40.^3^ Cost of treatment parameters for the model were modeled and validated from primary research at two private hospitals in Tamil Nadu, India. Private hospitals were chosen as the research focus because it is estimated that 80% of India’s outpatient care and 60% of India’s inpatient care is provided in the private sector.^32^ In Tamil Nadu, patients have a notable preference for private facilities, with as many as 90% of patients choosing private care for certain conditions.^33^ Probabilities were gathered from literature searches and informed by expert opinion in some cases. Health outcomes were measured in disability-adjusted life years (DALY), which is a composite measure that accounts for both years lived with a disability and early death. Disability-adjusted life years were discounted at 3%. Cost-effectiveness comparisons and determinations were made by calculating an incremental cost-effectiveness ratio (ICER), which is a ratio of net cost per DALY averted.

Cost parameters were derived from primary investigation at a rural private hospital. Costs were modeled for three case presentations: severe envenomation, mild envenomation, and no envenomation. For each case presentation, expert interviews and literature searches were conducted to identify required services, and the hospital fee structures were used to value the services and arrive at modeled cost parameters for the decision tree. Service fees were used as a proxy for treatment cost.

To validate the modeled cost data, empirical cost data were collected at a second private facility: an urban teaching hospital. At the urban hospital, financial records were available. Records were searched for all patients presenting with a snakebite diagnosis from 2008 to present. Seventy-one records were identified, and cost data were recorded as the direct cost to the patient, categorized by service provided. All costs were collected in Indian rupees and converted to 2015 USD using the commercial market conversion rate in May 2015.^34^ No corresponding medical outcomes were available. These data were used to calculate a mean cost of snakebite treatment, which was inflated to account for a 20.4% deficit between patient payments and total costs at the urban hospital.

The modeled and empirical cost averages differed by less than 1.5%. Because of this similarity, modeled costs were used as model parameters, because they allowed for disaggregation by severity (Supplemental Appendix 1). Sensitivity analyses were used to analyze uncertainty in cost parameters.

### Analytic approach.

The model was constructed to compare the two treatment strategies: 1) antivenom and supportive care and 2) an antivenom/adjunct combination strategy with supportive care. To account for potential intervention opportunities in a prehospital setting, the model begins at the time of snakebite. All subsequent costs and health outcomes were ascribed to the terminal branches of the tree and used for calculating outcome measures.

**Figure 1.**
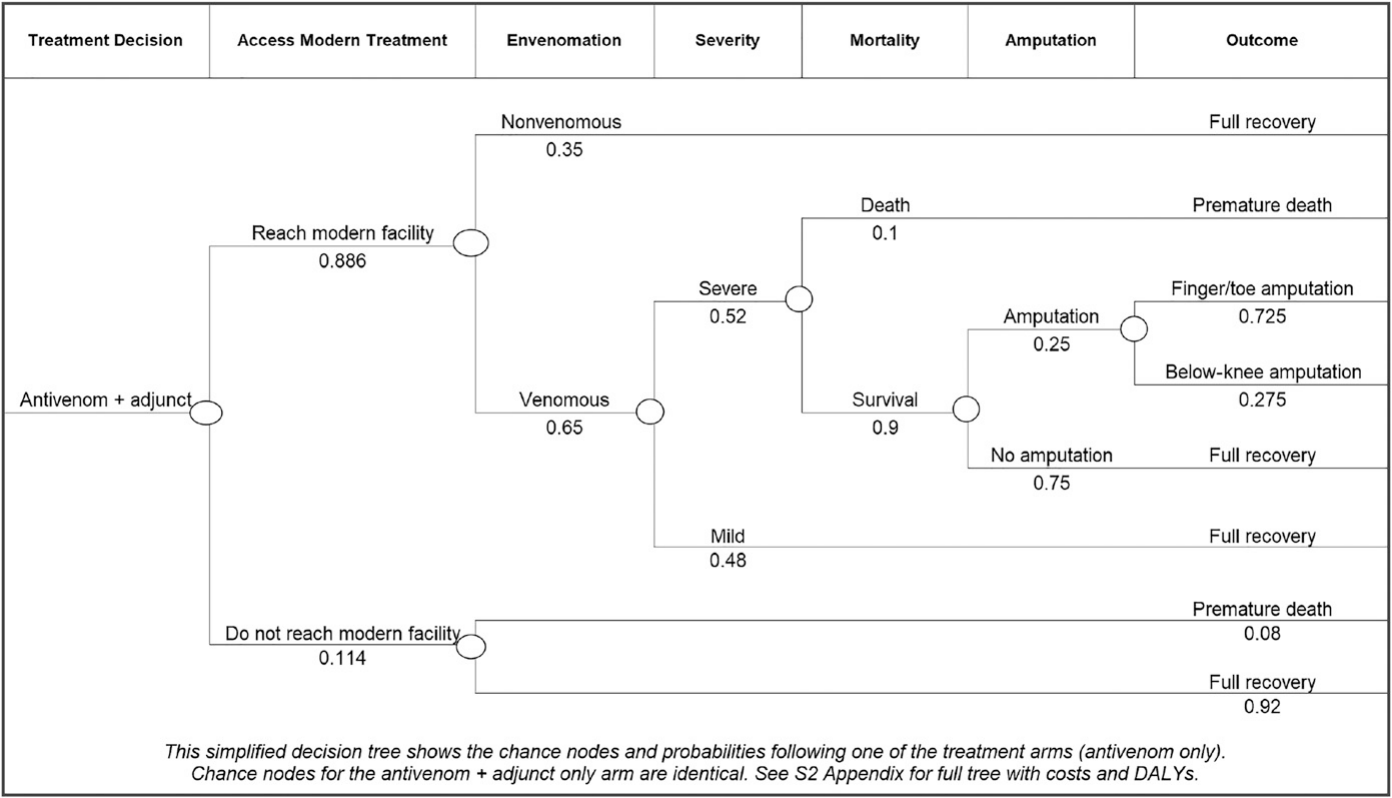
Simplified decision tree model. One treatment arm (antivenom + adjunct) with all subsequent chance nodes and probabilities. Amputation was chosen for this example because direct costs were known. Please see Supplemental Appendix 2 for the full decision tree.

The decision tree, shown simplified in Figure 1 and in full in Supplemental Appendix 2, was designed to categorize snakebite patients in one of three outcome branches: no envenomation, mild envenomation, and severe envenomation. Cases of “no envenomation” occur after a bite from a nonvenomous snake or after a “dry bite” from a venomous snake—when no clinically significant amount of venom is injected—and are treated with overnight hospitalization to rule out envenomation. Mild envenomation is characterized by clinical signs of envenomation, such as local tissue necrosis and pain, and requires treatment with antivenom, antibiotics, and several days of hospitalization. Severe envenomation is characterized by either kidney failure or the loss of breathing function due to paralysis. Severe envenomation is treated with large doses of antivenom, intensive care unit (ICU) supportive care, kidney dialysis and/or mechanical ventilation, and hospitalization for more than 1 week. Only severe envenomation carries the potential for lifelong disability due to limb amputation. Mortality and disability are most closely associated with 1) accessing treatment at a modern health facility, and 2) complications of supportive care in severe cases.^9,10^ Therefore, two corresponding parameters were varied between treatment branches: the percent of patients reaching a modern health facility and the percent of cases that are severe. Early treatment has a well-established effect on both of these outcomes,^9,10^ and any new treatment strategy should reduce prehospital mortality and the severity of cases once patients have reached the hospital.

### Model parameters.

Epidemiological parameters were derived from existing research on snakebite epidemiology (Table 1). This group of parameters does not depend on treatment strategy and was therefore held constant across each branch of the decision tree. It was important to model the percent of snakebites that are venomous because a large proportion of snakebites do not result in envenomation. Posthospital mortality and incidence of amputation were held constant across treatment branches because these probabilities are conditional on first being classified as a severe case.

**Table 1:** Model parameters

Parameter	Low	Base case	High	Sources
Cohort size	–	100	–	–
Discount rate	–	3%	–	–
Epidemiological parameters				
% Venomous snakes	32.5%	65%	97.5%	35*
Mortality following treatment (in severe cases)	5%	10%	15%	3,35–38
Incidence of any amputation (in severe cases)	12.5%	25%	37.5%	1,2,21,37,39
Incidence of finger/toe amputation (if any amputation)	50.75%	72.5%	94.25%	38,39
Mortality without treatment	0.8%	7%	15.2%	3,36
Intervention effectiveness				
% Accessing modern health facility (antivenom only)	79%	88.6%	96%	3
% *Increase* in accessing modern health facility (antivenom/adjunct)	1%	7%	11%	Assumption
% Severe cases (antivenom only)	50.4%	63%	75.6%	33,40*
Absolute % *Reduction* in severe cases (antivenom + adjunct)	3.9%	13%	22.1%	Assumption
Costs				
Supportive treatment cost of severe envenomation (not including AV)	$1,077	$2,153	$3,230	Primary investigation
Supportive treatment cost of mild envenomation (not including antivenom)	$253	$506	$760	Primary investigation
Supportive treatment cost of no envenomation	$50	$101	$151	Primary investigation
Cost of finger/toe amputation	–	$174	–	Primary investigation
Cost of below-knee amputation	–	$522	–	Primary investigation
Cost of antivenom (antivenom only)	$144	$287	$431	Primary investigation
Cost of antivenom (antivenom + adjunct)	$144	$287	$431	Primary investigation
Cost of adjunct	$157	$313	$470	Assumption
Cost of adjunct distribution/storage (per patient treated)	$1.31	$2.61	$3.92	Assumption
Disability weights				
Below-knee amputation	0.064	0.164	0.264	41,42
Finger/toe amputation	0.005	0.02	0.035	41,42

Base-case values, data sources, and ranges used in the probabilistic sensitivity analysis.

* These base-case values took various expert opinions into account in addition to published literature.

The base-case scenario is the combination of the most likely probabilities assigned to each parameter. For all parameters, base-case probabilities were assigned values within the range reported in literature. For probabilities associated with the new treatment strategy, informed assumptions were used in the base-case scenario. These assumptions were designed to be realistic and conservative. A combination treatment strategy was assumed to yield modest improvements in outcomes over antivenom alone. In the base-case scenario, we hypothesize that the percent of patients reaching a modern health facility in the combination therapy arm increases from 89% to 96% (7% increase compared with standard of care), and the percent of cases classified as severe versus mild in the combination therapy arm decreases from 63% to 50% (13% decrease compared with standard of care).

Because the actual price of individual adjunct strategies is not known, the base-case was purposely set at an extremely high estimate and varied from 50% to 150% in the probabilistic sensitivity analysis (PSA). To set up the base-case scenario, hypothetical new adjunct therapeutic strategies were chosen at the randomly generated cost of $313—higher than the cost of antivenom in the study setting (Tamil Nadu). Although we expect the cost of adjunct therapeutic or preventive strategies to be significantly less than the current cost of antivenom, we purposely chose a high cost because low cost of adjunct therapeutic strategies are likely to be realized only after scale-up in production and dissemination. It is yet unknown which, and how many, types of adjunct strategies will be required to reduce envenomation severity. See also Supplemental Appendix 1 for more details of cost parameters.

Disability weights for chronic disabilities due to amputation were taken from the Global Burden of Disease (GBD) study, but we did not include analysis of loss-of-function of limbs in this analysis—we included only amputation because the cost of amputation is more predictable for an exploratory study such as this one. Because of the large differences between the WHO disability weight update in 2004 and the GBD 2010, a range was used to account for both numbers in the sensitivity analysis. The base-case disability weight for below-knee amputation (0.164) was taken from the GBD 2010, and the base-case disability weight for finger/toe amputation (0.02) was an assumption informed by several related disability weights reported in the 2004 update and the GBD 2010.^41,42^

### Outcome measures.

The primary outcome measures are the thresholds for treatment effectiveness and cost. These thresholds are defined as the increase in effectiveness and net cost at which a combination strategy becomes the more cost-effective option. As a secondary outcome measure, we report the ICER, which is the ratio of incremental costs per DALY averted. To compare an antivenom/adjunct combination strategy with antivenom alone, the ICER was calculated based on the difference of costs and DALYs between these two strategies.

### Sensitivity analysis.

To analyze the uncertainty in model parameters and identify which parameters have the greatest effect on the outcome measure, several types of sensitivity analysis were used in this study. First, one-way sensitivity analyses were used to identify thresholds of interest. For example, the percent of cases classified as severe was varied while holding all other variables constant at base-case values to identify thresholds for both cost-effectiveness and cost savings. The same analysis was carried out for the cost and effectiveness of an adjunct therapeutic.

Next, two scenario analyses were conducted. The first scenario examined the cost-effectiveness of the combination strategy compared with antivenom alone, assuming the additional adjunct has no effect on the severity of envenomation and only affects prehospital mortality. The second scenario analysis varied the cost of the adjunct across a range of potential values and recorded the corresponding ICER (versus antivenom only) of each cost estimate.

Finally, a PSA was conducted using @Risk, Version 7 (Palisade, Ithaca, NY). This analysis used prior distributions for each model parameter. During model simulation, values were randomly chosen from each prior distribution and used to calculate ICER values. The simulation was conducted for 10,000 iterations.

For all parameters, prior distributions were defined by the beta distribution, with minimum and maximum values reported in Table 1. This is in accordance with ISPOR recommendations for parameters from scarce or unverified sources (such as expert opinion).^43^ Because costs were modeled from hospital fee schedules, they were subject to the same degree of uncertainty as health parameters. Beta distributions are feasible and conservative estimates of both health and cost parameters.

## RESULTS

### Threshold analysis.

**Figure 2.**
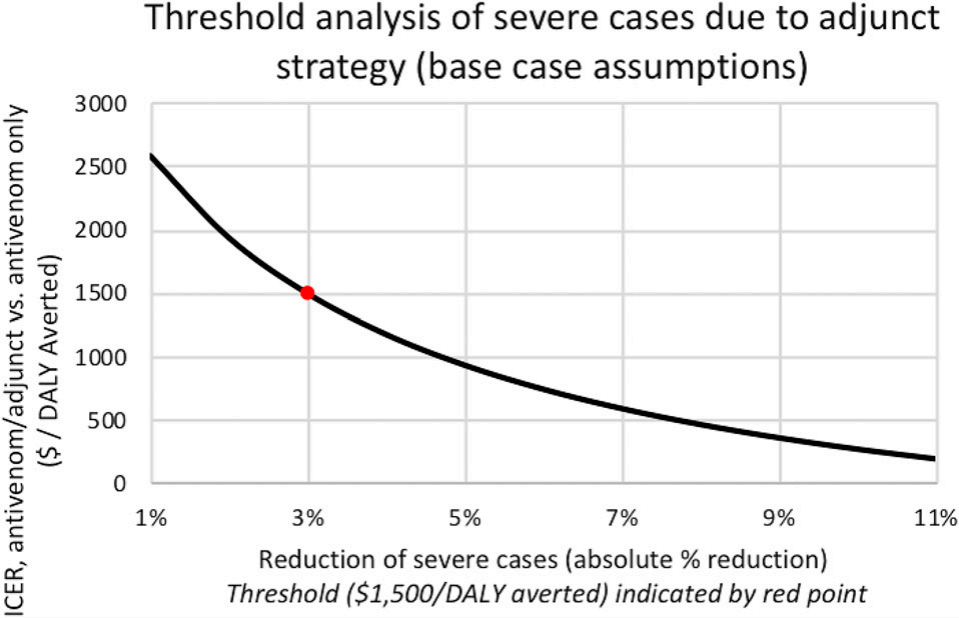
Threshold analysis of severe cases (primary result) on the ICER using an antivenom/adjunct approach. The cost-effectiveness threshold ($1,500 per DALY averted) is reached with a 3% absolute reduction and is indicated by a red point. The antivenom/adjunct strategy averted an additional 0.28 DALYs per person compared with antivenom only. The ICER of interest to this study (antivenom/adjunct combination strategy vs. antivenom alone) had a value of $68 per DALY averted. DALY = disability-adjusted life year; ICER = incremental cost-effectiveness ratio. This figure appears in color at www.ajtmh.org.

Two deterministic threshold analyses were conducted on the parameters associated with adjunct effectiveness: percent of patients reaching hospital treatment and percent of patients with severe envenomation. These analyses were conducted at base-case values by varying each parameter independently of the others. Threshold analysis showed that the combination strategy would be cost-effective (defined as averting one DALY per $1,500 investment—equivalent to India’s *per capita* GDP at the time of study)^44^ if any strategy costs less than $1,608 per treatment regimen, assuming base-case effectiveness (13% reduction in severe cases versus mild cases and 7% increase in patients reaching the hospital). The threshold for cost savings was reached when adjunct therapy costs dropped below $252 per regimen. Analysis indicated cost-effectiveness if combination treatment could reduce the percent of severe cases by 3% (Figure 2).

For results disaggregated by health and cost contributions, see Table 2. Note that all costs are incurred and saved in the first 2 weeks of the time horizon, while health effects continue to contribute to the analysis throughout the 43-year time horizon (due to chronic condition).

**Table 2:** Results of base-case analysis (secondary results)

Intervention	DALYs per person	DALYs averted per person	Net cost per person	Costs added per person	ICER vs. prior table entry
Antivenom + supportive care	1.23	–	$1,046	–	–
Antivenom/adjunct + supportive care	0.95	0.28	$1,065	$19	$68

DALY = disability-adjusted life year; ICER = incremental cost-effectiveness ratio. At base-case values, the antivenom only strategy resulted in a total cost per person of $1,046 and the antivenom/adjunct strategy resulted in a total cost per person of $1,065 (an increased investment of $19 per person).

### Scenario analysis.

**Figure 3.**
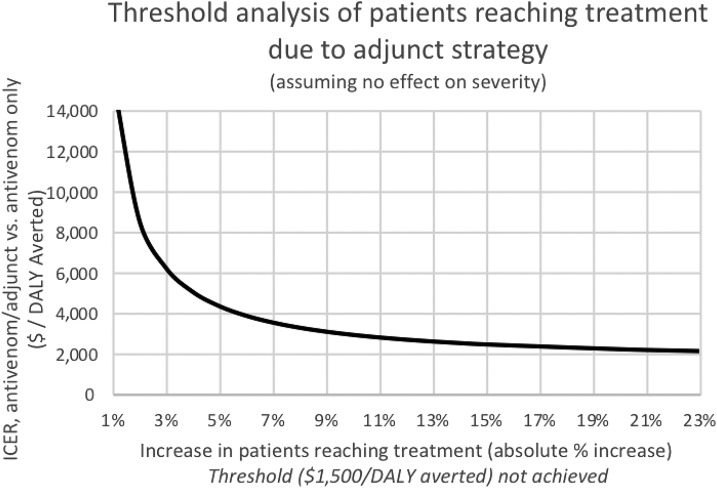
Threshold analysis of patients reaching treatment (assuming no decrease in severe cases). The effect on the incremental cost-effectiveness ratio (ICER) resulting from varying the percent of patients reaching treatment because of the addition of an adjunct. This analysis assumes an adjunct has no effect on the percent of cases that are severe. As patients reaching treatment increases, the cost-effectiveness improves; however, this does not permit the strategy to reach $1,500 per disability-adjusted life year (DALY) averted even when 100% of patients reach treatment (23% absolute increase).

Our first scenario analysis was conducted to determine 1) the cost-effectiveness threshold for reduced severity, *assuming no increase in patients reaching treatment*, and 2) the cost-effectiveness threshold for increased numbers of patients reaching treatment, *assuming no reduction in severe cases*. Assuming no increase in patients reaching treatment, an absolute reduction in severe cases by 3% would achieve cost-effectiveness (Figure 2, above). Assuming no decrease in the proportion of severe cases, increasing the percent of patients reaching treatment improves the cost-effectiveness of the treatment; however, because of the high cost of that treatment, varying this parameter alone does not cause the ICER to reach the cost-effectiveness threshold even when the percent of patients reaching treatment is set to 100. The results of this analysis are shown in Figure 3.

The results of the second scenario analysis, which varies the cost of the adjunct strategy through several possible values, are presented in Table 3.

**Table 3:** Results of scenario analysis (cost to individuals of adjunct per regimen) cost savings *per person* are average savings for an individual treated with antivenom and an adjunct strategy, vs. antivenom alone

Cost of adjunct	Cost savings per person
$1	$78
$5	$77
$10	$75
$15	$74
$20	$72
$30	$69
$100	$47
$252	$0

In this model, all costs and savings are incurred in the first 2 weeks following snakebite.

### Probabilistic sensitivity analysis.

**Figure 4.**
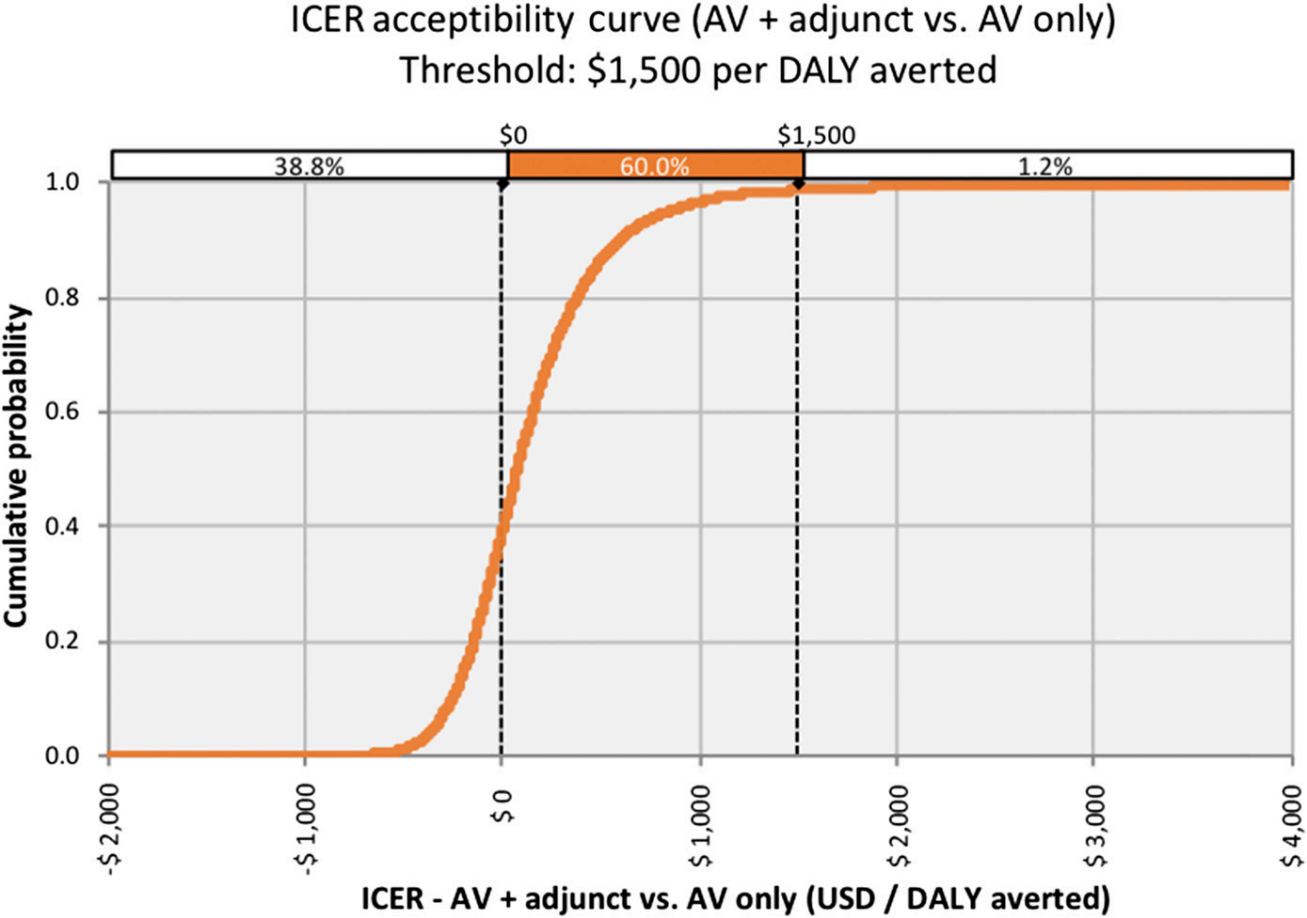
Incremental cost-effectiveness ratio (ICER) acceptability curve: the cumulative probability of the ICER after simulation with 10,000 iterations. The cost-effectiveness threshold was set at $1,500 per disability-adjusted life year (DALY) averted above which the intervention was not considered cost-effective. This figure appears in color at www.ajtmh.org.

The PSA resulted in 1.2% non-cost-effective iterations, 60% cost-effective iterations, and 38.8% dominant iterations (simultaneously savings costs and increasing health benefits) (Figure 4). Although negative ICERs are not always dominant, in this analysis, negative ICERs could only be produced by increasing health benefits and decreasing costs.

**Figure 5.**
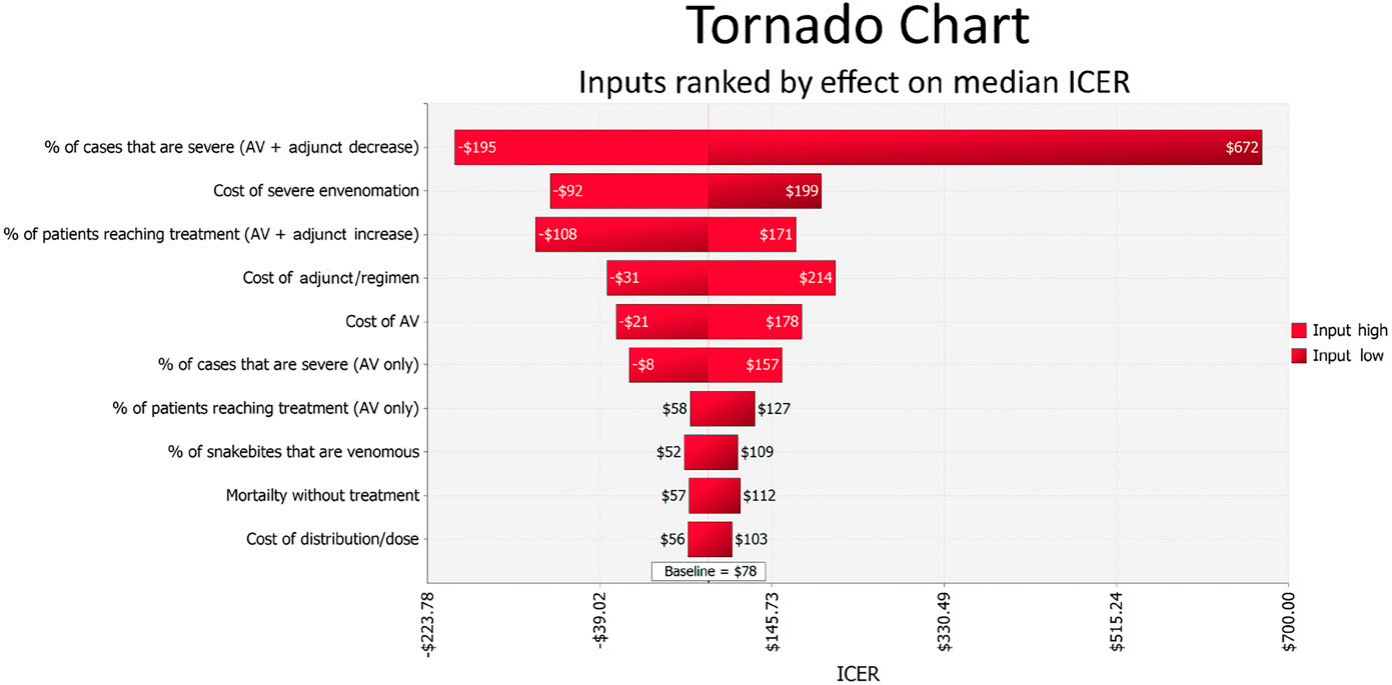
Tornado chart: the effect of the 10 model parameters that have the greatest effect on the median incremental cost-effectiveness ratios (ICER). Results are based on probabilistic sensitivity analysis with 10,000 iterations. This figure appears in color at www.ajtmh.org.

Probabilistic sensitivity analysis indicated that reducing the severity of envenomation has the greatest effect on the overall cost-effectiveness of any additional intervention. See Figure 5 for a tornado graph showing the 10 parameters with the greatest effect on the ICER.

## DISCUSSION

Our analyses suggest that an absolute reduction of severe cases by just 3% resulting from implementation of any adjunct strategy to antivenom for snakebite treatment could be highly cost-effective or even cost saving, *regardless of whether there was an increase in the number of patients reaching treatment*. We used the “very cost-effective” threshold recommended by the WHO, even though there are alternative models for this type of determination.^45^ At mid-range cost, even if an adjunct preventative or treatment strategy shows moderate effectiveness, its implementation would be cost saving when less than $252 and even an investment of $10 will result in $75 savings per patient with only a 3% reduction in the incidence severe cases.

Our sensitivity analysis indicates that a reduction in severe cases is likely to have the greatest impact on cost-effectiveness. The high costs associated with hospitalization in the ICU, combined with additional costs of dialysis, mechanical ventilation, and other intensive management, are greater determinants of cost-effectiveness than the direct cost of antivenom or any proposed adjunct (e.g., a field antidote, bite-mitigating outerwear, or pressure immobilization). If an adjunctive therapeutic could even modestly avert respiratory paralysis, tissue damage, or acute kidney injury, a large portion of treatment costs could be averted while simultaneously averting mortality and lifelong disability.

Though actual bills and treatment packages from two hospitals in Tamil Nadu were reviewed, this study is limited by sufficient access to a broader sample of real-world data. In addition, elements of this analysis are implicitly speculative. Also, hospitals where data were collected did not have long-term health outcomes for the same patients, so it was not possible to associate costs with health outcomes without elements of cost modeling. Supplemental Appendix 1 shows how the cost scenarios were modeled, along with weighted averages using probabilities in the decision tree. Although the possibility of significant estimation error still exists and was accounted for in the sensitivity analysis, the similarity between the two cost estimates suggests the reliability of the results.

Incidence of snakebite and bite severity, as well as the quality, delivery, and timeliness of medical care varies significantly by region. This study’s real-world data are specific to one area of southern India, and the base-case scenario may not be generalizable to other regions. We focused on private health-care providers, which comprise most treatment delivery in India.^46^ Nevertheless, parameter ranges were wide enough to encompass a range of possible real-world scenarios, and estimates were generally conservative, even for southern India. Thus, the main outcomes of this study, including the cost-effectiveness of an adjunct-based approach and the importance of averting hospitalization in the ICU, should be generalizable to any region with a significant burden of snakebites.

This model showed a relatively weak relationship between the proportion of patients reaching a hospital and the cost-effectiveness of an intervention. This is because patients seeking treatment following a bite incur much greater costs than patients who do not receive any modern treatment, regardless of whether their prognosis was improved by implementation of effective adjunctive strategies.

Further epidemiological studies should seek to adopt a community-based approach to understand the true burden of snakebites in rural regions. Economic research should seek to estimate costs using a prospective micro-costing approach when feasible. Otherwise, retrospective economic studies should attempt to gather health outcome data in combination with patient expenditures and collateral losses.

This study suggests that decreasing the severity of snakebite cases has the greatest effect on overall cost-effectiveness and that investment in prevention and prereferral treatments should be considered in programmatic responses to this neglected tropical disease. Although the theoretical implementation of adjunct strategies appears to be highly cost-effective, many questions still need to be answered to reduce the global burden of snakebites. We did not assess whether and how different interventions would be accepted by key stakeholders (e.g., individual citizens, physicians as well as local, regional, and national governments and governing organizations) in terms of concept and cost. For example, if an efficacious, reasonably priced adjunct therapeutic were made available in rural settings, would it be adopted by affected populations and by health-care providers? At what price would it be commercially viable while achieving the goal of wide accessibility? How would it be implemented in a prehospital setting? These and many other questions require further qualitative, quantitative, and mixed-method investigations. Answers are more likely to be achieved with a coordinated response to the global burden of snakebite.

## Supplementary Material

Supplemental appendices
