# Supplementary material for: Snakebite: An Exploratory Cost-Effectiveness Analysis of Adjunct Treatment Strategies
Source: Am J Trop Med Hyg. 2018 Jun 4;99(2):404–12. doi: 10.4269/ajtmh.17-0922 (PMC6090346; doi:10.4269/ajtmh.17-0922)
Supplement: Supplementary file 3 [file tpmd170922.SD3.pdf]

Supplemental Appendix 1

Cost tables: items, unit costs, and totals for all model costs

Supplemental Appendix 2

Full decision tree, including costs and disability-adjusted life years associated with each arm
